# Early-stage investigators’ experiences with an National Institutes of Health Pilot Award Program

**DOI:** 10.1017/cts.2025.2

**Published:** 2025-01-10

**Authors:** Cyleste C. Collins, Jacqueline Dolata, Elodie Nonguierma, Mona Shediac-Rizkallah, Ashwini R. Sehgal, J. Daryl Thornton

**Affiliations:** 1 School of Social Work, Cleveland State University, Cleveland, OH, USA; 2 Center for Health Equity, Engagement, Education, and Research, The MetroHealth System, Cleveland, OH, USA; 3 Case Western Reserve University School of Medicine, Cleveland, OH, USA

**Keywords:** Early-stage investigators, qualitative, health disparities, mentoring, grants management, learning

## Abstract

**Background:**

Academic-community research partnerships focusing on addressing the social determinants of health and reducing health disparities have grown substantially in the last three decades. Early-stage investigators (ESIs), however, are less likely to receive grant funding from organizations like the National Institutes of Health, and we know little about the facilitators and barriers they face on their career journeys or the best ways to support them and their community research partnerships. This study examines ESIs’ experiences with a program that funded and supported their community-partnered pilot health disparities research.

**Methods:**

Fourteen ESIs from five cohorts of pilot investigators participated in in-depth focus groups between April 2020 and February 2024. Two reviewers independently identified significant quotes and created codes. Thematic analysis was used to develop relevant themes.

**Results:**

The overarching theme was that the program was a launch pad for the ESIs’ research careers. Four distinct sub-themes contributing to the launch pad theme were: (1) ESI Growth & Adaptation; (2) Community and Support; (3) The Value of Collaboration and Partnership; (4) Need for Effective Mentorship. The results suggest the program offered ESIs and community partners substantial, unique support and resources, but challenges remained.

**Conclusions:**

Future programs helping ESIs who conduct community-engaged research to launch their research careers should consider implementing tailored support while offering strategies to eliminate or reduce institutional barriers, including strengthening mentoring.

## Introduction

Academic-community partnerships conducting research addressing the social determinants of health and reducing health disparities have grown substantially in the last three decades [1]. Research has identified many approaches for enhancing collaborative research capacity among academic and community partners [2]. Recent studies examine partnership models’ (e.g., investigator-led vs. agency-led) benefits and challenges; using small grants to build academic-community partnerships for community-initiated health projects; funding community-based organizations to partner with researchers to disseminate findings relevant to their communities; and funding community-based organizations to build collaborative research capacity [2–5]. Building and enhancing collaborative partnerships has the potential for developing a synergy in which “community and academic partners accomplish more together than could be accomplished alone.” [6, p. 435]^.^ Building a synergy that translates into action has the potential to reduce or eliminate health disparities.

While academic/community research partnerships are essential, nurturing such partnerships requires intensive time and energy, especially when working with early-stage investigators (ESIs). ESIs tend to be disadvantaged in receiving National Institutes of Health (NIH) funding, especially women or researchers of color. Men and more experienced investigators receive most research funding [7,8]. NIH data from 2021 show that while women ESIs submitted 46.7% of applications, they were less likely to receive funding compared to their male counterparts, even though their applications had slightly higher discussion rates [9]. Black ESIs, meanwhile, made up 4% of applications (compared with 2% for established) and had a lower discussion and funding rate than other ESIs [9].

In U.S. higher education, the myth of meritocracy assumes that success is based solely on individual effort and academic merit. However, this overlooks the structural barriers that disproportionately affect underrepresented minority scholars, particularly those who are also ESIs. One such barrier is epistemic exclusion, a phenomenon where the knowledge and contributions of these scholars are undervalued or ignored, further compounding their challenges in securing recognition, funding, and career advancement [10]. The problem has important consequences, particularly for advancing the careers of ESI women and researchers of color in science and academia, where they tend to be underrepresented [11]. This study focuses on the experiences of mostly women ESIs participating in a pilot program.

Although there have been efforts to increase women’s representation at higher academic ranks, such efforts have resulted in little change. Women hired into academia face many challenges, including receiving lower pay, less startup funds, waiting longer for promotion, and being less likely to be a primary investigator on large, prestigious grants, such as those funded by NIH. They are also less likely to be awarded grant funding, have fewer publications as lead author, and are less likely research to have their research cited [7,12–18]. COVID-19 worsened these numbers, stalling women’s progress as they were less likely than men to submit manuscripts, (hampering their progress toward tenure or promotion), and dropped out of academic roles at higher levels. Research reports that during that time, many women were “considering leaving their positions or reducing hours… especially female faculty with children” [14,19, p. 436]. Even before the COVID-19 pandemic, careers in research in academia were not highly attractive to women. A recent study examining 35 years of data from more than half a million graduates from 134 medical schools in the U.S. found women were less likely than men to be promoted above the assistant professor level in academic roles [17]. Women also leave postdoctoral positions in larger numbers than men, and among those who attend graduate school, fewer pursue careers in higher education or research [20,21]. It is likely, then, that women ESIs need more support now than they have in decades.

## Program context, description, and aim

ESIs who are women or are otherwise underrepresented face several disadvantages, and they therefore may benefit from intensive support. This study describes work from the Investigator Development Core program of a grant funded by the National Institute on Minority Health and Health Disparities U54 mechanism, “Involving Communities in Delivering and Disseminating Health Disparity Interventions” at the Center for Health Equity, Engagement, Education, and Research (CHEEER) in Cleveland, Ohio. The program awarded up to $50,000 per pilot project annually to five cohorts of ESIs (3-4 each) from 2018 to 2023. The program focused on building underrepresented ESIs’ capacity to engage in partnerships with communities to conduct high-quality community-engaged health disparities research. The program design and implementation have been described elsewhere [22]. This study describes the ESIs’ experiences with the program.

## Methods

### Design

In-depth focus groups were chosen as the ideal approach for this research because the individuals involved were from the same program and were acquainted as fellow program participants. We sought to understand the diversity of experiences among the attendees, the extent to which they had similar experiences, and the possibility that hearing other participants’ feedback would spark participants to share aspects of their own experiences [23].

### Interview guide

The interview guide was developed by the program’s external evaluator who had extensive experience in CEnR and had worked with CHEEER’s community programing as an external evaluator for eight years. The evaluator developed the interview guide with feedback and input from the project director. The interview guide began with an explanation that the focus group was intended to learn about the interviewees’ experiences with the project. The first question prompted each participant to discuss their project and its current phase. Participants were asked to describe their research partners, how closely they had worked together, and whether they had worked with them before. They were then asked what they felt the most distinctive or memorable part of the program was, and how their program experiences had affected them personally and/or professionally. They were asked about the extent to which they felt the project would help move them toward future studies. Follow-up questions included: “To what extent would you say your experience with this project would make you more/less/equally likely to participate in a similar project again?” and “What would you tell a friend or colleague who might be interested in applying for the program about what to expect?” The questions then proceeded toward what recommendation(s) they would make about how to improve the program, feedback on the program’s staff, impressions of the community-based research network meetings (including asking about the meetings’ usefulness, and topics the meetings should address). The interview concluded by inviting participants to share any additional thoughts or experiences they had.

### Recruitment & procedures

The program director informed the ESIs about the focus groups verbally during the announcements at a monthly Community-Based Research Network (CBRN) meeting which the ESIs and members of the community attended. ESIs were reminded about the focus groups via email. For each cohort, one or more focus group dates were identified, and the program’s external evaluator e-mailed invitations to all ESIs. The invitation outlined the interview’s purpose, identified the focus group’s date and time, and provided a link to an online consent form approved by a university-based institutional review board. The focus groups were conducted via Zoom and lasted approximately one hour. The external evaluator facilitated the focus group. All ESIs from the five cohorts were invited to participate in the focus groups. Two did not respond to the e-mailed focus group invitation, and two did not attend due to scheduling conflicts. Fourteen ESIs participated in one of five focus groups, one in April 2020 (cohorts 1 & 2), two in March 2023 (cohorts 3 & 4), or two in February 2024 (cohort 5). All study protocols were approved by a university-based institutional review board, and ESIs who attended provided informed consent. No incentives were offered for their participation. The focus groups were recorded using a voice recorder and a professional transcriber transcribed the audio.

### Analysis and procedures to establish qualitative data trustworthiness

The focus groups were analyzed using thematic analysis following its stepwise process [24]. First, analysts (between two and five for each transcript) read and familiarized themselves with the transcripts, highlighting significant quotes and developing codes describing their significance. The analysts met to discuss their codes, including which were similar (i.e., they highlighted the same things) and different, and discussed how to group the codes and develop themes. The analysts then reviewed and defined the themes. All qualitative data were managed in Atlas.ti (Version 9.1.3).

We employed several efforts to establish data trustworthiness, including establishing credibility through prolonged engagement and persistent observation, analyst triangulation, peer debriefing, and maintaining an audit trail to establish confirmability. Four analysts had been engaged with the program on multiple levels for multiple years prior to this study, which allowed for prolonged engagement and persistent observation. Through its work with community members in similar agencies who were conducting research, the team had deep knowledge of the study context. Analyst triangulation involved having multiple analysts code and analyze quotes to avoid investigator bias. Peer debriefing included discussing findings and interpretation with a colleague familiar with the program and CEnR. Detailed notes and records used in tracking the analysis process established an audit trail.

## Results

### Participants

The ESIs’ demographic characteristics are displayed in Table 1. All but one participant was a woman. The participants worked in schools of medicine, nursing, and social work. The ESIs’ research projects were at least one year in but were all at different stages. Data collection was complete for all but one, and three had participated in some initial dissemination efforts, including conference presentations and journal manuscript submissions.

**Table 1.** Early-stage investigators’ demographics

Characteristic	ESIs (N = 18)	(%)
Gender		
Women	17	94.4
Men	1	5.5
Race/Ethnicity		
White	14	77.7
Black	2	11.1
Asian	1	5.5
Middle eastern	1	5.5
Faculty status		
Assistant professor	10	55.5
Postdoctoral fellow	5	27.8
Affiliated clinical faculty	3	16.7
School/hospital/department		
Medicine	13	33.3
Nursing	3	16.6
Social work	2	11.1

### Overarching theme: ESIs viewed the program as a career launchpad

**Figure 1.**
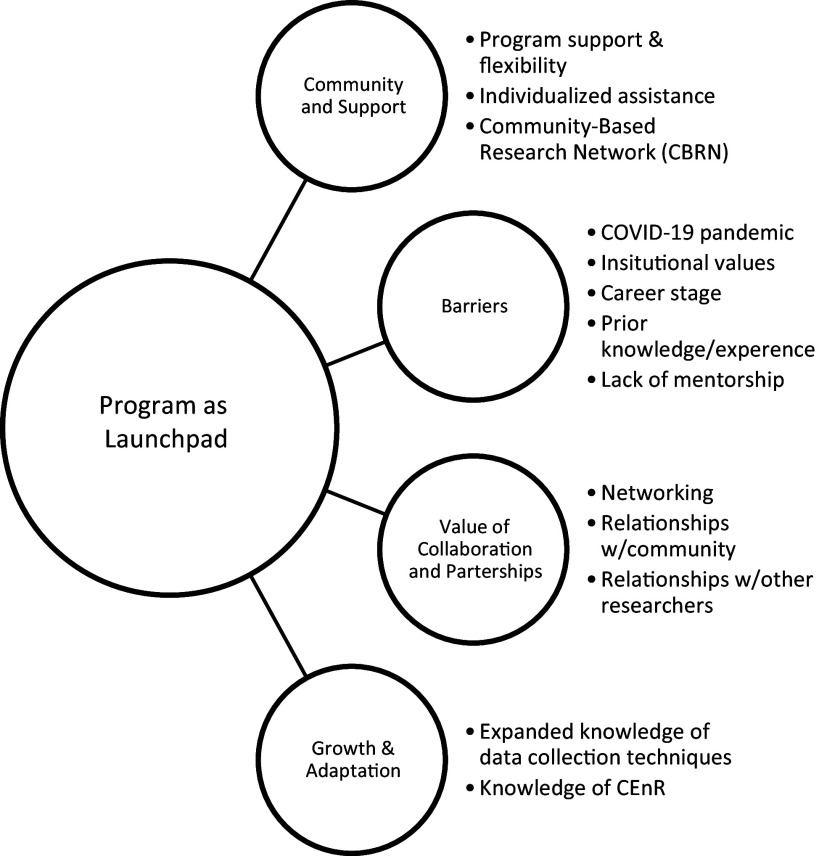
Theme and sub-themes.

The overarching theme that the program had been an important career launchpad encapsulated ESIs’ experiences (see Fig. 1). A quote from one reflected the theme.

It really helped launch my research. I’m so grateful in that way. I had written for a number of …NIH R03 grants and things like that and didn’t have much luck, and then being able to submit to something like this and having conversations with people from the [program staff] helping me hone in my research questions more. From there, I received three other grants.

Other ESIs talked about submitting proposals to NIH after the program. Although two said their proposals did not make it to the discussion phase, they were undaunted and planned to move forward “with additional ideas” anyway. The sub-themes below were elements of the overarching theme (see Table 2). While the first three sub-themes describe ways participating in the program helped launch their careers (growth and adaptation, community and support, and the value of collaboration and partnership), the last sub-theme describes mentorship barriers ESIs experienced during the program.

**Table 2.** Themes, descriptions, and representative quotations

Sub-themes	Description	Representative quotation
Sub-theme 1: Early-stage investigator growth & adaptation	Working with research participants	“Our focus groups were so powerful, not realizing the emotions that were tied with it. So as we’re going back and reviewing some of the data…“Wow.” It just makes me feel like I’m not doing enough.”
Sub-theme 2: community & support	Monthly Community-Based Research Network meetings	“Getting exposure to what everybody else was working on. It’s good for idea-generating.”
	Connecting/interacting with people from other disciplines	“It was just the opportunity to …to interact with people from other disciplines, which I don’t often get to do.”
Sub-theme 3: the value of collaboration and partnership	Community partner relationship/breaking out of academic silos	“It was really interesting to hear not only [partner’s] side of things, but also I ended up interviewing his employees …and meeting other people in the community that had the same goal in mind.”
	Community dissemination activities	“We had a celebration …I had a sheet that went through our major findings …and generated conversation and discussion around “What do we think are some workable solutions to some of the problems that have been highlighted?” …it was really fun to put our heads together and spend time with the whole team.”
Sub-theme 4: need for effective mentorship	Distinct from general and specific program support; need for more individualized guidance	“I just felt like I was sort of thrown into the deep end and figured out how to swim and waded through most things by myself”

**Sub-Theme 1. Growth and Adaptation.** One way the program served as a launchpad was by helping the ESIs grow by developing new skill sets. Several ESIs said the pilot grant was their first, and others said their research knowledge and experiences had been limited before the project. One shared their background:

My PhD was in quantitative big data methodology and running numbers, … I didn’t have a very strong basis in qualitative research. …My initial proposal was a mixed-method study. I had to convert to qualitative, because that’s the best that I could do with the time that I had, and …this grant helped me to strengthen my ability to understand qualitative research, work through it and have support.

The ESIs also talked about developing skills in administering grants, working with budgets, hiring research assistants, and managing their time to balance their other work responsibilities with research. They also said they developed skills in working directly with organizations and their communities, especially understanding their priorities and goals.

**Sub-Theme 2. Community and Support.** A second major way the ESIs feel the program helped them launch their careers was through the program’s organizational infrastructure, which offered informational support, tangible resources, and other assistance. The CBRN was an important, built-in support network, composed of community members, community organization representatives, and academics knowledgeable of and interested in CEnR and health disparities. The ESIs said the CBRN helped them navigate community-engaged research challenges. One said, “The community and the monthly meetings were the best part about it and getting exposure to what everybody else was working on. It’s good for idea-generating.”

One ESI said:

This is my first grant, so I don’t have a lot to compare it to. …All my prior research was more on my own with mentors, but not really part of a network like this. I found it extremely helpful…to get an idea of how others were approaching certain issues or problems, and what programs and initiatives were happening in the community.

Another said it was important to gather and discuss their work, and “you could tell we are all facing similar challenges. Sometimes it was also a space to brainstorm a little bit about how to move forward.” The program staff, ESIs said, were pivotal in asking them to bring up their biggest challenges and strategies for working through them. One ESI said, “I found that to be really helpful… like troubleshooting and learning how other people are dealing with certain issues that come up.” Sharing ideas, updates, and processes from “people that drank the community-engaged research Kool-Aid” was described as “inspirational” and helped them think about “these larger structural things… how can we advocate on a higher level to address some of these barriers?”

Changes in the ESIs’ personal lives served as a barrier for several ESIs, and job changes, illness, moves, and babies’ births affected their career trajectories. One ESI explained her experience.

My personal situation changed completely and as part of the move and birth of the baby, my career track turned 180, or 90 degrees, and went in a completely different direction and doesn’t include any research because my new job doesn’t have the capacity to provide any other type of work. My research career, at least for now, is put on hold or over, … but that’s definitely not a reflection of my experience or of the grant itself.

The supportive infrastructure and flexibility carried through to flexible timelines, as five ESIs were on family leave after having babies during their project period. Despite the barriers ESIs’ life changes imposed, the ESIs said the program staff continued to help them through and adjusted timelines according to their needs. The ESIs were grateful for the program’s support and flexibility, especially during COVID. One said,

We all learned a lot. …I really appreciate having the flexibility within this grant to navigate what that looked like to have the support and resources of people who are like-minded in health disparities research and community-engaged approaches and working together to figure out ways to navigate that.

This comment talked about the barriers COVID posed to data collection and the program’s support in helping them figure out workarounds. The ESIs said the program offered both informal (tailored support for specific situations) and formal support (e.g., the CBRN).

**Sub-Theme 3. The Value of Collaboration and Partnership.** The ESIs discussed the importance of building trusting partnerships with their community partners and networks with other community organizations and ESIs. They also emphasized how the program infrastructures and the CBRN supported and facilitated those relationships. Regarding developing relationships with fellow ESIs, including those from other disciplines, one ESI said, “I connected with a really good qualitative researcher who I think I’ll probably work with in the future.”

The ESIs said their strong community partnerships were crucial in connecting with research participants. While some participants had worked with their community partners before and others had not, all felt the program strongly supported the partnerships. The trust the ESIs built with their community partners also strengthened their connection to the communities with which they were working, reflected especially in the qualitative data they collected. The qualitative data were a key way ESIs communicated their trustworthiness to their community partners. Several ESIs talked about being moved by their study participants’ willingness to share their stories and lived experiences” with them. One said they learned their participants wanted “to pave the way” for others in the future and contribute to making conditions better. Another said their findings indicated that not enough was being done to help the population being studied, and it motivated them to do more. Other ESIs noted the vulnerabilities of their populations and their privilege to learn from them, experiences described as “eye-opening.” One ESI said:

Working with the [participants] and see[ing] them going through the process of utilizing the intervention and hearing their feedback. Our focus groups were so powerful, not realizing the emotions that were tied with it. So, as we’re going back and reviewing some of the data, it’s just like “Wow.” It just makes me feel like I’m not doing enough for them.

Another ESI said,

As someone who is a physician and has been in this medical silo, …I found that to be really rewarding and it was really interesting to hear people’s perspectives, what people knew and didn’t know, and to truly be able to have a better sense of the community.

Another ESI said it was exciting that her community partnership was changing practice due to the study’s findings. As a physician, she was able to link her community partners with patients in the hospital where she worked. She said she was “very excited about …the potential to bring additional awareness” to the interconnections between medical care and community and believed the work would result in “a richer collaboration …because of what we started.” Another ESI said, “We didn’t know at the time, but the ability to take something that we’d started with, run with it and grow was amazing.”

Important to trust-building with community partners was identifying and utilizing partners’ strengths. ESIs talked about learning about and from one another and identifying the skills each could contribute to the project. One ESI said flexibility was important, as early in the partnership, research roles had to be revisited to utilize partners’ skills most efficiently.

Initially some of the people that were put on the team didn’t quite work out the way that we thought that they would. They had different skills than we expected… We were able to, in the moment kind of reassign roles and make sure that we were taking the best advantage of everyone’s skills.

The ESI said their partner’s “institutional culture allowed for that conversation to happen.” Another ESI echoed how important it was to work with partners’ strengths. “You know you have to identify some of the strengths of some of your partners, and for those that are not on board, kind of redirect them so that they don’t feel like you’re totally abandoning them or not valuing what they have to contribute.” While role reassignment could have been a source of tension, the supportive organizational environment avoided it.

Not all partnerships were positive, however. One ESI spoke about a different experience they had with their partner due to the pandemic.

It started out really well…Then of course COVID happens and I feel like… everyone’s attention all of a sudden shifted…It was really hard to keep in contact after that because [partner] was obviously really overworked… and had much higher priorities.

This specific challenge was unique and directly tied to COVID and was an outlier in our sample.

**Sub-Theme 4. Need for Effective Mentorship.** While overall, the ESIs said they had positive experiences with the program, they mentioned some areas in which the program could improve in the future (Table 3 has a complete list). The ESIs discussed barriers hindering their project’s flow including lacking grant management knowledge, gaps in mentorship, and conflicts with partners.

**Table 3.** Areas of improvement

Needs/areas of improvement	Representative quotation
Individual, informal, focused mentoring/coaching	“It would be very beneficial to do some really good mentorship …to say, “But do you really have enough time to do this?” and also “Is the scale of your project appropriate for the 10%?””
Grants management training	“An area that I feel I’m still really lacking is how to actually manage a grant and know where, what ifs, and how things are moving. I feel like that aspect is lacking and nobody teaches us.”
Budget training	“There will definitely be a lot of us who have no experience figuring out how to manage the money and how that works. I had no idea.”
Clear expectations	I mean all of us came with this pilot study of about three pages that we wrote, but …“Is this really a health disparities project… did we capture the right components? …Are we focused on the right outcome mechanism?” …Where is that feedback? …I’m a junior person, so expecting some type of feedback as I’m moving along within this process. …There were different perceptions of what the overall program was offering.
Contingency plans/community partner follow-through	“It would’ve been helpful to have some backup, someone …to really sit down and be like “How are we gonna work through this?”…Sit down and go over what are the next steps to do, if the person doesn’t answer your emails.”
Clarification of mentors’ roles	“Maybe that’s some room for growth, to clarify what is that role of a mentor and what kind of commitment would that entail from the mentor.”
Attending to sustainability	“I think it would actually be beneficial if like during the planning phase there was more focus on sustainability for some projects.”
“Pilot” suggests smaller/less demanding project	“I worry that the title pilot indicates or somehow conveys that it’s smaller than it is.”
Clarity, flexibility around time	“There are huge delays, and that’s not acknowledged enough in community-based research, that you need that first year of building a foundation, the second year of data collection, and then the third year at least of dissemination and action type of pieces. That all got condensed into a one-year timeline that kept getting extended, but we also didn’t know if it was gonna be extended until right before.”

The ESIs discussed the need for more grant support, especially in developing and managing grant budgets. They focused on lacking guidance in understanding grant management basics, such as tracking budget balances and knowing grant terminology. “An area that I feel I’m still really lacking is how to actually manage a grant and know where, what ifs, and how things are moving. I feel like that aspect is lacking and nobody teaches us.” This gap in knowledge highlighted the need for effective mentorship and support in navigating grant administration.

I had to have my mentor help me, walk me through the budget that I was constructing. I was like “What is indirect costs?”…There was some minimal guidance on the internet, on their webpage, that you could go to, but it didn’t really break it down to the basics.

While some ESIs had strong mentors, others felt hesitant or reluctant about asking for assistance and felt that a certain level of independence was expected of them. ESIs felt there were some grant-related things they thought they should know but did not.

I just felt like I was sort of thrown into the deep end and figured out how to swim and waded through most things by myself, but I would reach out to different people, if I was stuck. … [Program staff member] was always one of the go-to people and then she would point me maybe to somebody else who could help me navigate something like IRB.

This ESI did not remember who her mentor was for the project, and there was no memorandum of understanding (MOU) to outline a mentor’s responsibilities. She went on to suggest an MOU might be helpful as she had several experiences with mentors who didn’t fulfill the mentorship role effectively. Formalizing the relationship with an MOU would clarify mentors’ obligations to their mentees and ensure accountability and regular check-ins. “Everybody’s so busy. All professors have so much on their plates, too, and so they kind of sign off on a lot of things.” Such structural changes, she said, might help address the common issue of mentors’ limited time and availability.

Other ESIs felt unprepared to deal with conflicts with their community partner and did not want to appear like they needed excessive assistance. Some ESIs likened this to being “shy” or worrying about being “pushy and coercive” by asking for help. “It’s almost like handholding, so I don’t know if at some point you have to be grown and do it” a quote that may reflect their early-career status.

## Discussion

This study aimed to gain a better understanding of ESIs’ needs and experiences within a pilot community-engaged research partnership program. Overall, our findings highlight both the positive impacts of a program designed to support ESIs in conducting community-engaged health disparities research and helping with the barriers they face. We found that the ESIs felt the program helped them launch their research careers and helped them grow and adapt to changing environmental conditions. They also felt the community and support the program provided were essential and valued their community partners’ collaboration and partnership. The ESIs valued their research experiences, recognized their partners brought unique skills, resources, and expertise, and felt their work was richer because of that. The ESIs also valued the program’s specific organizational infrastructure, highlighting the strong, attentive staff support, and the helpfulness of career-building specific resources.

Despite the program’s assistance, ESIs faced some persistent challenges, including institutional barriers. ESIs cited additional needs for effective mentorship on both the individual level and program levels. While the pilot funds were a great start, ESIs needed assistance with very specific aspects of their research projects, at the application phase (e.g., developing budgets), and during project implementation (e.g., developing IRB applications, recruiting and hiring research assistance, recruiting participants, obtaining participant incentives, etc.) to successfully carry out their research. ESIs commented that meeting times for activities and the CBRN did not always work for them, and some said they would have preferred to meet in person, however, they admitted finding a time that would work for everyone would be challenging, especially given their clinical responsibilities.

### Navigating academia: mentoring and supporting ESIs in CEnR

Our participants’ positive experiences with the program and enthusiasm about its supportive environment as well as the transformative potential their research findings had for community practice, echo sentiments from prior literature [25]. Having a supportive infrastructure for ESIs in CEnR partnership can provide an important foundation for beginning research careers and has the potential for increasing equity and reducing disproportionality in federal funding. The value of a supportive infrastructure is consistent with the National Academies of Sciences, Engineering, and Medicine’s emphasis on the importance of the organizational domain in training health disparities professionals [25]. Our findings also align with existing literature focused on the growth of academic-community collaborations in addressing health disparities and exploring models to enhance collaborative research capacity, emphasizing community engagement [1,2].

Although much research on CEnR indicates how important it is to equalize power and knowledge in CEnR, it also tends to assume academic partners are research experts, and the learning curve will be primarily around how to work with community partners [6]. Our findings indicate, however, that ESIs are simultaneously learning about both being a good academic partner within CEnR and how to lead an independent research project. ESIs’ need for mentorship reflected a tension between feeling they should be self-reliant but also needing support. ESIs’ felt under-prepared for handling, not necessarily academic research’s rigors, but its logistics fully independently. The myth of meritocracy is rooted in U.S. values of individualism value “making it” despite barriers and without assistance. Academic structures, with their invisible rules, concepts, and definitions, set people up to think they should know what to do, and that others’ assistance should be unnecessary. These structures, which are often implicit, can be a method of academic gatekeeping, excluding particular groups [10]. Similarly, academic and research language is often specialized, jargon-filled, and not understandable without specialized training. Research argues that promoting equity in academic spaces requires demystifying academia by making the invisible visible and that lacking knowledge is developmentally appropriate and not a deficit [26], a sentiment with which our findings agree.

While McKay & Devlin’s work focuses on students from low socioeconomic backgrounds and ESIs are among the most highly educated people in society, working for elite institutions, *they still needed help* in understanding the intricacies of the research process and research steps [27]. Epistemic exclusion, implemented through formal and informal hierarchies, defines the types of knowledge that are deemed most “valuable”, and hinders underrepresented scholars’ academic advancement [10]. For example, when academic institutions do not support and/or recognize CEnR in tenure and promotion policies, this communicates that the work is seen as less rigorous and/or important [28]. Demystification requires explicitly supporting those who are in the early stages and/or are underrepresented in academia. Developing, maintaining, and strengthening mentoring programs may be essential tools for leveling the playing fields for new investigators and ESIs. Supportive infrastructures are thus crucial for ESIs conducting CEnR, as such support may increase equity and reduce disproportionality in federal funding [25]. Although this study’s program had built-in support with the CBRN, which provided ESIs with essential support around their learning about working effectively with community, they needed more than the CBRN could offer. Strong, individualized, and tailored mentoring could help fill the gap [29].

### Limitations

Several limitations should be considered in interpreting the results. First, the small sample of ESIs, most of whom were White women cannot fully represent the diversity of experiences among ESIs engaged in health disparities research. The small sample also means the findings might not be generalizable to all ESIs or CEnR partnerships outside of the program. Second, the focus groups were conducted at three different points in time (although conducted at similar time points in the ESIs’ project trajectories), which likely influenced the ESIs’ perspectives. Although the first and second cohort interviews took place a few weeks after pandemic lockdowns began and their projects were mostly completed, the third and fourth cohorts were significantly more challenged by the pandemic given their projects were in their earlier stages. Such changes and challenges might have also affected institutional policies (e.g., remote work requirements), shifts in community priorities based on the pandemic, and ESIs’ career development possibilities. All of these may limit the applicability of the findings to non-pandemic circumstances. Third, as with any qualitative study relying on self-reported data, there is potential for bias. ESIs could have provided socially desirable responses, or they might not have fully disclosed challenges due to concerns about confidentiality being interviewed in a focus group. Fourth, because the study focused on ESIs within a specific program but working with people nested in institutions, and knowing institutional culture and support systems vary widely, the results might not reflect the experiences of ESIs in other institutional contexts. Finally, this study does not track the long-term outcomes of the ESIs’ careers or the sustainability of their community partnerships. Future research following the ESIs would provide a more comprehensive understanding of the program’s impact over time.

### Implications

Despite the study’s limitations, our findings have implications for programs and future research. The earlier findings were used to improve later cohorts’ experiences. The program immediately implemented pre-application consultations and resources to address concerns the first two cohorts of ESIs raised about the mentoring and support they needed. The first two cohorts did not have formal mentors, so formal mentors were required to sign on for subsequent cohorts. However, this did not fully solve the problem. Good mentors were hard to find, and this is consistent with past research. One study examining Harvard-affiliated academic researchers’ training and mentoring needs found that about half of junior faculty were interested in having a mentor for their CEnR, however, few academic researchers were willing to serve as one [30] Taken together, our findings and previous literature suggest that mentor development and encouragement are both necessary. Past research indicates high-quality mentorship and/or sponsorship and systems supporting women in academia must recognize and support caregiving responsibilities [19]. In the CEnR realm, this is particularly important, as “few academic institutions invest in the infrastructure that can enable faculty members to work in and with communities” [25, p. 88]. Thus, ESIs engaged in CEnR may need additional resources and support.

Our findings suggest that both internal and external institutional policies are important in supporting underrepresented ESIs conducting CEnR. Internally, institutions need to establish infrastructure distinct from mentoring, including strong administrative support and extended project timelines, beyond the typical one-year pilot, to ensure ESIs can fully develop their projects. Externally, funders should prioritize tailored mentoring and long-term relationship-building between academic and community partners, rather than funding short-term projects. Creating communities of practice in which ESIs share success stories and challenges while engaging in co-learning would contribute to ESIs’ projects and partnerships’ long-term sustainability.

Future efforts like this one should begin with the assumption that ESIs–particularly those underrepresented in research and academia– are in the early stages of their careers which means they are new to writing grant proposals, developing and administering research budgets, and partnering with the community. This newness, combined with underrepresentation, including mentors who are similar to them, may mean they need considerable assistance in both areas. Based on our findings, future programs should include efforts that cultivate community culture, including skill development, infrastructure support, support for fostering relationships, including mentorship, and working to remove institutional barriers to enhance ESIs’ experiences, including ensuring flexible and realistic project timelines that account for normal life challenges. Policymakers should ensure that these factors are built into program design to provide systemic support for underrepresented ESIs.

Beneficial strategies include ensuring ESIs have mentors, and/or strengthening mentoring relationships through formal MOUs and/or incentives. Mentors should be experienced researchers who are invested in the ESI and not in name only. Mentors should be intentionally and actively engaged in the professional growth of the ESI. Training on mentor expectations could address many of these gaps, as would creating formal mentor-matching programs between previous ESIs and newer cohorts. Additionally, offering concrete policy support for caregiving responsibilities would help retain diverse ESIs in the field. Providing training on expectations and mentors’ roles might effectively address this issue. Previous program participants/ESIs could also be formally matched with ESIs as mentors. Similarly, senior researchers with a strong understanding of ESIs’ needs could be part of a larger strategy of developing supportive, sustainable faculty groups. Program developers and policymakers can use these findings to inform policies and develop programs for supporting ESIs engaging in CEnR.

Acknowledging this study’s methodological limitations, we urge further research to explore additional facilitators and barriers to ESIs’ success in CEnR and to develop robust support mechanisms adaptable to a variety of institutional settings. If, as the present study suggests, ESIs need strong mentoring and support, then future research needs to explore how best practices in recruiting and retaining effective reluctant mentors. We need to know more about what characterizes “good” mentors. Creating CEnR mentor training programs and institutionalizing support through policies could help equip ESIs with what they need to succeed. CEnR mentor training programs could be developed to give ESIs what they need. Additionally, and crucially, academic institutions need to make a commitment to ensuring they have strong mentors to help ESIs succeed. High-quality mentorship must recognize and support caregiving responsibilities, if relevant. As more research identifies ESIs’ specific needs, institutions can consider concrete ways of investing in infrastructure (e.g., training, mentoring, and promotion policies) to support CEnR at the institutional level. Institutionalized support is an important factor in helping maintain long-term CEnR partnerships [31] Extending mentorship beyond short-term programs and supporting long-term professional development through administrative support and relationship-building would help the retention and success of underrepresented ESIs in CEnR.

One strategy includes research development (RD) which involves helping ESIs assess their research-related career goals and planning for how to achieve those goals [27]. However, our work clearly indicates much more specific assistance beyond RD is needed. Investing in supportive programs in science may address women’s underrepresentation in academia especially early in their careers [19] and promote equity in academic spaces. Such work has the potential of helping to democratize research knowledge and processes, which may go a long way toward achieving equity [27].

## Conclusion

This study enhances our understanding of ESIs’ experiences in CEnR partnerships and the support they need in developing research agendas addressing health disparities through such partnerships. By understanding ESIs’ experiences and needs, we can better assist them in overcoming challenges and furthering their research careers. These findings are promising, and future research should continue to seek to discover ways to support ESIs traditionally underrepresented in research and academic settings. Investing in programs that support ESIs through specific mentoring and encourage collaborative partnerships will create more equitable and effective research with real-world impacts on health disparities.

## References

[1] Ortiz K , Nash J , Shea L , et al. Partnerships, processes, and outcomes: a health equity-focused scoping meta-review of community-engaged scholarship. Annu Rev Public Health. 2020;41(1):177–199. doi: 10.1146/annurev-publhealth-040119-094220. 31922931 PMC8095013

[2] Kegler MC , Blumenthal DS , Akintobi TH et al. Lessons learned from three models that use small grants for building academic-community partnerships for research. J Health Care Poor Underserved. 2016;27(2):527–548. doi: 10.1353/hpu.2016.0076. 27180693 PMC5554883

[3] Collins C , Dolata J , Pike E , Sehgal A. Increasing research capacity in community organizations: findings from the community research scholars initiative. Eval Program Plann. 2023;96:102189. doi: 10.1016/j.evalprogplan.2022.102189. 36436308 PMC9801679

[4] Gopalan G , Bunger AC , Powell BJ. Skills for developing and maintaining community-partnerships for dissemination and implementation research in children’s behavioral health: implications for research infrastructure and training of early career investigators. Adm Policy Ment Health. 2020;47(2):227–243. doi: 10.1007/s10488-019-00930-5. 30863918 PMC6742583

[5] Theurer J , Pike E , Fischer RL , Collins C. The community research scholars initiative: a mid-project assessment. Clin Transl Sci. 2015;8(4):341–346. doi: 10.1111/cts.12286. 26073663 PMC4553124

[6] Coombe CM , Chandanabhumma PP , Bhardwaj P , et al. A participatory, mixed methods approach to define and measure partnership synergy in long-standing equity-focused CBPR partnerships. Am J Community Psychol. 2020;66(3-4):427–438. doi: 10.1002/ajcp.12447. 32744781 PMC7772255

[7] Hechtman LA , Moore NP , Schulkey CE , et al. NIH funding longevity by gender. Proc Natl Acad Sci. 2018;115(31):7943–7948. doi: 10.1073/pnas.1800615115. 30012615 PMC6077749

[8] National Institutes of Health. research grants: awards by gender and percentage to women, 2022, Accessed October 20, 2022, https://report.nih.gov/nihdatabook/report/171

[9] Lauer M. More early stage investigators supported in FY 2021. extramural NEXUS July 18, 2022, https://nexus.od.nih.gov/all/2022/07/18/more-early-stage-investigators-supported-in-fy-2021/.

[10] Settles IH , Jones MK , Buchanan NT , Dotson K. Epistemic exclusion: scholar(ly) devaluation that marginalizes faculty of color. J Divers High Educ. 2020;14(4):493–507. doi: 10.1037/dhe0000174.

[11] National Academies of Sciences E, Affairs P and G, Committee on Women in Science, E, etal. Factors that drive the underrepresentation of women in scientific, engineering, and medical disciplines, Promising Practices for Addressing the Underrepresentation of Women in Science, Engineering, and Medicine: Opening Doors. National Academies Press (US); 2020, Accessed May 1, 2024, https://www.ncbi.nlm.nih.gov/books/NBK555386/ 32134611

[12] Burstin HR , Arora VM. Gender disparities in journal citations—Another metric of inequity in academia. JAMA Netw Open. 2021;4(7):e2114787. doi: 10.1001/jamanetworkopen.2021.14787. 34213562

[13] Chatterjee P , Werner RM. Gender disparity in citations in high-impact journal articles. JAMA Netw Open. 2021;4(7):e2114509. doi: 10.1001/jamanetworkopen.2021.14509. 34213560 PMC8254129

[14] Kibbe MR. Consequences of the COVID-19 pandemic on manuscript submissions by women. JAMA Surg. 2020;155(9):803–804. doi: 10.1001/jamasurg.2020.3917. 32749449

[15] Lo Sasso AT , Armstrong D , Forte G , Gerber SE. Differences in starting pay for male and female physicians persist; explanations for the gender gap remain elusive. Health Aff Proj Hope. 2020;39(2):256–263. doi: 10.1377/hlthaff.2019.00664. 31967925

[16] Oliveira DFM , Ma Y , Woodruff TK , Uzzi B. Comparison of national institutes of health grant amounts to first-time male and female principal investigators. JAMA. 2019;321(9):898–900. doi: 10.1001/jama.2018.21944. 30835300 PMC6439593

[17] Richter KP , Clark L , Wick JA , et al. Women physicians and promotion in academic medicine. N Engl J Med. 2020;383(22):2148–2157. doi: 10.1056/NEJMsa1916935. 33252871

[18] U.S. Department of Labor. Families first coronavirus response act: Employee paid leave rights. https://www.dol.gov/agencies/whd/pandemic/ffcra-employee-paid-leave, Accessed February 10, 2021.

[19] Davis PB , Meagher EA , Pomeroy C , et al. Pandemic-related barriers to the success of women in research: a framework for action. Nat Med. 2022;28(3):436–438. doi: 10.1038/s41591-022-01692-8. 35177858

[20] Martinez ED , Botos J , Dohoney KM , et al. Falling off the academic bandwagon. Women are more likely to quit at the postdoc to principal investigator transition. EMBO Rep. 2007;8(11):977–981. doi: 10.1038/sj.embor.7401110. 17972894 PMC2247379

[21] UNESCO. UNESCO science report: towards 2030. United Nations Educational, Scientific and Cultural Organization. 2015;1–817. https://unesdoc.unesco.org/ark:/48223/pf0000235406

[22] Collins C , Thornton D , Nonguierma E , Dolata J. Building as you fly: Launching and evaluating a pilot program to mentor early-stage investigators in community-engaged research. Poster presented at: American Public Health Association Annual Conference and Expo, Atlanta, GA; November 2023.

[23] Morgan DL. Basic and Advanced Focus Groups. Thousand Oaks, CA: SAGE Publications, Inc; 2019.

[24] Braun V , Clarke V. Using thematic analysis in psychology. Qual Res Psychol. 2006;3(2):77–101. doi: 10.1191/1478088706qp063oa.

[25] National Academies of Sciences, Engineering, and Medicine. A framework for educating health professionals to address the social determinants of health 2016, National Academies Press (US);, http://www.ncbi.nlm.nih.gov/books/NBK395983/, Accessed May 20, 2021. 27854400

[26] McKay J , Devlin M. Uni has a different language … to the real world: demystifying academic culture and discourse for students from low socioeconomic backgrounds. High Educ Res Dev. 2014;33(5):949–961. doi: 10.1080/07294360.2014.890570.

[27] Droegemeier KK. Demystifying the Academic Research Enterprise: Becoming a Successful Scholar in a Complex and Competitive Environment. Cambridge, MA: MIT Press; 2023.

[28] Marrero DG , Hardwick EJ , Staten LK , et al. Promotion and tenure for community-engaged research: an examination of promotion and tenure support for community-engaged research at three universities collaborating through a clinical and translational science award. Clin Transl Sci. 2013;6(3):204–208. doi: 10.1111/cts.12061. 23751026 PMC3852909

[29] Manson SM. Early-stage investigators and institutional interface: importance of organization in the mentoring culture of today’s universities. AIDS Behav. 2016;20(2):304–310. doi: 10.1007/s10461-016-1391-0. PMC4995131 27044483

[30] DiGirolamo A , Geller AC , Tendulkar SA , Patil P , Hacker K. Community-based participatory research skills and training needs in a sample of academic researchers from a clinical and translational science center in the northeast. Clin Transl Sci. 2012;5(3):301–305. doi: 10.1111/j.1752-8062.2012.00406.x. 22686211 PMC3374155

[31] Ward M , Schulz AJ , Israel BA , Rice K , Martenies SE , Markarian E. A conceptual framework for evaluating health equity promotion within community-based participatory research partnerships. Eval Program Plann. 2018;70:25–34. doi: 10.1016/j.evalprogplan.2018.04.014. 29894902 PMC6077092

